# Underlying Mechanisms for Growth Promotion by Low-Concentration Single Salt and Alkali Stresses and Growth Inhibition by Combined Salt-Alkali Stress in *Quercus mongolica*

**DOI:** 10.3390/microorganisms14030547

**Published:** 2026-02-27

**Authors:** Fan Huang, Xinrui Wu, Laixue Zou, Te Li, Tongbao Qu

**Affiliations:** College of Forestry and Grassland, Jilin Agricultural University, Changchun 130118, China; 18365105429@163.com (F.H.); 16688203008@163.com (X.W.); 13943136294@163.com (L.Z.); 15140122381@163.com (T.L.)

**Keywords:** *Quercus mongolica*, saline-alkali stress, rhizosphere microbiome, hormesis, plant–soil–microbe interactions

## Abstract

Soil salinization is a global ecological issue that severely constrains forest tree growth and ecological restoration. The salt-alkali stress response mechanisms of *Quercus mongolica*, a key temperate forest species in China, remain unclear. A two-factor pot experiment was conducted using NaCl (0, 50, 100, 200 mmol·L^−1^) and NaHCO_3_:Na_2_CO_3_ (1:1; 0, 50, 100, 150 mmol·L^−1^). Plant traits, soil properties, and enzyme activities were measured. Furthermore, high-throughput sequencing revealed that microbial responses enhanced network cooperation under 100 mmol·L^−1^ salt stress and improved network stability under 50 mmol·L^−1^ alkali stress. These responses also upregulated resistance genes and increased soil enzyme activities. This activation of seedling antioxidant and osmotic adjustment systems was directly associated with an increase in growth parameters. Under combined stress, however, soil environment deterioration and microbial network disruption, along with reduced key soil enzyme activities, resulted in an insufficient defense system to counteract reactive oxygen species (ROS) accumulation, thereby reducing growth parameters. The study found that low-concentration individual salt or alkali stress promoted *Quercus mongolica* seedling growth, while combined stress was associated with significant inhibition. This study refines the theoretical framework for non-salt-tolerant trees and establishes a basis for determining their survival thresholds in saline-alkali soils.

## 1. Introduction

Soil salinization is a global ecological issue [1,2], with over 1.381 billion hectares of saline-alkali land worldwide expanding annually by 1% to 2% [3]. Soil salinization impairs the physiological functions of forest trees through ionic toxicity, osmotic stress, and nutrient immobilization [4], thereby inducing photosynthetic inhibition, membrane damage, and growth retardation [5], and ultimately markedly compromising the capacity for seedling establishment and survival [6]. In the face of this ecological challenge, investigating plant responses to saline-alkaline stress has become a major research focus.

Single salt stress, such as NaCl, can significantly reduce stem diameter and plant height in *Lagenaria siceraria*, as well as decrease seedling biomass in *Triticum aestivum* and *Gossypium hirsutum* [7,8,9]. It generally induces a decline in photosynthetic performance, reduction in chlorophyll content, and aggravation of membrane lipid peroxidation in leaves [10,11]. Concurrently, levels of stress-related substances, such as antioxidant enzyme activity, proline (Pro), and abscisic acid, are observed to increase markedly in plants [12,13,14]. Certain species, such as *Viola tricolor*, may exhibit transient growth promotion under low salt concentrations, but this shifts to inhibition as stress intensifies [15]. Ultimately, the suppression of morphological development and disruption of physiological metabolism contribute to significant growth inhibition in plants. Compared to salt stress, alkaline stress imposes a dual pressure of high pH and high Na^+^, typically exerting stronger inhibitory effects on plants [16]. For instance, it significantly suppresses the growth and root development of *T. aestivum* seedlings and various *Avena sativa* cultivars and interferes with the absorption and translocation of mineral elements, such as phosphorus and sulfur, in *G. hirsutum* [17,18,19]. Alkaline stress also elevates antioxidant enzyme activities and Malondialdehyde (MDA) content in the leaves of plants such as *Prunus humilis* and *Trachyspermum ammi*. In contrast, species such as *Hordeum jubatum* and *Leymus chinensis* exhibit increases in chlorophyll content and antioxidant enzyme activities, or alterations in nitrogen metabolite accumulation and soluble sugar (SS) content, respectively [20,21,22,23]. These complex and divergent responses highlight the distinctive impact of alkaline stress. However, in natural habitats, soil salinization and alkalization processes frequently co-occur, imposing more complex combined salt-alkali stress on plants; however, studies on such combined stress remain limited [24,25].

Currently, elucidating the rhizosphere microbe-mediated adaptation processes is a key step in deciphering plant response mechanisms under salt-alkali stress [26,27]. This salt-alkali stress alters the physicochemical properties of rhizosphere soil, such as pH and nutrient availability, creating a distinct microenvironment for microorganisms [28]. This subsequently induces significant shifts in the structure and function of the microbial community [22,29], which in turn affects the activity of key soil enzymes [30,31,32]. This microbe-mediated rhizosphere process ultimately influences key physiological states of plants, such as photosynthetic performance [33], antioxidant systems, and biomass allocation [34], by modulating their nutrient and water uptake [35]. However, current research following the pathway of salt-alkali stress, the rhizosphere microbiome, and plant responses has primarily focused on herbaceous species [27]. Related investigations in woody plants remain relatively limited [36], with existing studies largely concentrating on physiological responses and molecular regulatory mechanisms under single salt stress [37]. Systematic comparative studies examining how salt stress, alkaline stress, and their combination differentially affect the multidimensional system of plant physiology, rhizosphere soil, and microbe interactions are still lacking.

*Quercus mongolica* is an important tree species with both ecological and economic value in northern forest regions [38]. Current research primarily focuses on its genetic breeding [39], physiological responses to abiotic stress [40], and growth-promotion mechanisms mediated by exogenous microorganisms [41]. While the influence of abiotic stress on rhizosphere microbial communities is extensively studied [42], preliminary observations from our study revealed an unusual physiological response of *Q. mongolica* under specific saline-alkali conditions, which deviates from the typical behavior of glycophytes. Whether salt stress, alkaline stress, and their combined effects regulate the reshaping of the rhizosphere microbial community of *Q. mongolica* through different pathways, and thereby differentially affect its stress adaptability, remains to be clarified. Therefore, this study analyzed the growth and physiological responses of *Q. mongolica* and the properties of its rhizosphere soil under different saline-alkali stress conditions. Key stress treatments were selected for in-depth microbiome analysis to achieve the following objectives: (1) to systematically clarify the specific effects of different stress types on *Q. mongolica* and its rhizosphere environment; (2) to reveal the potential mechanisms by which different stress types differentially reshape the structural and functional characteristics of the rhizosphere microbial network; and (3) to elucidate the role of plant–soil–microbe interactions in regulating the saline-alkali adaptability of *Q. mongolica*.

## 2. Materials and Methods

### 2.1. Plant Material and Experimental Design

This study was conducted at the Plant Basic Research Center of Jilin Agricultural University. The cultivation substrate was nutrient soil, which was prepared by mixing peat, perlite, and vermiculite at a volume ratio of 2:1:1. Initiated in June 2024, all test seedlings were grown from seeds collected from the campus of Jilin Agricultural University (125°24′28.8″–125°25′12″ E, 43°48′–43°48′57.6″ N). Based on pre-experimental results and target soil salinity-alkalinity gradients, three concentration levels were assigned to single salt stress and single alkali stress to cover both hormetic and inhibitory effects. The alkali stress was imposed using a 1:1 molar mixture of NaHCO_3_ and Na_2_CO_3_. For combined salinity-alkalinity stress, nine treatment groups were established using an orthogonal design, with detailed protocols provided in Table 1. Seedlings with consistent growth performance were transplanted into plastic pots filled with the test substrate for final establishment, and trays were placed at the bottom of the pots for the collection of leachate. In accordance with the preset soil salt content, expressed as a mass fraction for each treatment, salts and alkalis were precisely weighed, dissolved to form aqueous solutions, and evenly irrigated into each pot in three successive applications at a volume of 250 mL per application, with a 12-day interval between consecutive applications. During the experiment, soil moisture was replenished as needed to maintain a 50% water-holding capacity. Thirty days after the last stress treatment, plant growth parameters were measured, and mature intact leaves from upper shoots were collected for physiological index determination, with three seedlings randomly selected as samples per treatment and three biological repeats. Following sample collection, part of the leaf samples were reserved for chlorophyll content measurement, and the rest were weighed and stored at −80 °C for subsequent physiological analysis. Soil samples were collected from rhizosphere soil adhering to *Q. mongolica* seedling roots, placed in sealed plastic bags after impurity removal, and divided into two parts: one sieved through a 60-mesh sieve and stored at 4 °C for soil physicochemical properties and enzyme activity determination, and the other flash-frozen in liquid nitrogen for soil metagenomic sequencing.

### 2.2. Determination of Plant Growth-Physiological Indices, Soil Physicochemical Properties, and Enzyme Activities

Plant height increment was defined as the difference between the initial and final measurements from the stem base to the apical growing point, taken with a tape measure. Stem diameter increment was quantified as the difference between initial and final stem base diameters through an electronic vernier caliper. Total biomass was determined as the sample dry weight. Samples were first treated at 105 °C for 30 min for enzyme deactivation and then dried to constant weight at 75 °C before weighing. Chlorophyll content was measured by the ethanol extraction method. Superoxide dismutase (SOD), peroxidase (POD), and catalase (CAT) activities were assayed using the nitroblue tetrazolium (NBT) method [43], the guaiacol method [44], and ultraviolet spectrophotometry, respectively [45]. MDA content was determined by the thiobarbituric acid (TBA) colorimetric method [46]. SS, proline (Pro), and soluble protein (SP) contents were assayed through the anthrone colorimetric method, the acidic ninhydrin colorimetric method [47,48], and the Coomassie Brilliant Blue G-250 method, respectively [49].

Soil pH was determined using a Leici PHS-25 pH meter, and EC was measured using a Leici DDS-307A conductivity meter. Soil water content (SWC) was measured by the oven-drying method. Soil total organic carbon (TOC) content was quantified via the potassium dichromate oxidation-external heating method [50], and TN content was determined using an elemental analyzer [51]. Soil urease (S-UE), soil catalase (S-CAT), and soil sucrase (S-SC) activities were assayed by indophenol blue colorimetry [52], ultraviolet spectrophotometry [53], and 3,5-dinitrosalicylic acid (DNS) colorimetric method [54], and soil alkaline phosphatase (S-ALP) activity was determined via thep-nitrophenylphosphate (pNPP) colorimetry in a corresponding fashion [55].

### 2.3. Selection of Treatments for Metagenomic Analysis

Based on the growth response characteristics of *Quercus mongolica* seedlings to gradient concentrations in preliminary experiments, representative treatments were selected for metagenomic sequencing in combination with two-way ANOVA results. The analysis showed that salt stress and alkaline stress had significant main effects on growth and physiological indices, soil physicochemical properties, and enzyme activities (*p* < 0.05), with significant interactive effects observed for certain parameters under combined stress. Following this differentiated pattern of plant–soil responses, four treatments were selected for metagenomic sequencing—CK, S_2_, A_1_, and S_2_A_1_—with three biological replicates per treatment. Microbial community diversity data were compared using one-way ANOVA.

### 2.4. DNA Extraction, Library Preparation, and High-Throughput Sequencing

Soil metagenomic sequencing was performed by Novogene Co., Ltd. (Beijing, China). Briefly, 1 µg of genomic DNA per sample was randomly sheared to a target fragment size of approximately 350 bp using a Covaris ultrasonicator (Covaris, Inc., Woburn, MA, USA). Sequencing libraries were constructed using standard procedures, including end repair, A-tailing, adapter ligation, and PCR amplification [56,57,58]. Library size distribution and integrity were evaluated with a Fragment Analyzer system (Advanced Analytical Technologies, Inc., Ames, IA, USA). Libraries satisfying the expected size criteria were then precisely quantified using quantitative PCR (qPCR) to ensure an effective concentration exceeding 3 nM, which is a critical prerequisite for reliable library pooling [59] (He et al., 2025). Subsequently, qualified libraries were pooled at equimolar ratios according to their qPCR-derived effective concentrations and the desired sequencing depth. Equimolar pooling was conducted based on qPCR-determined concentrations and desired depth, followed by PE150 sequencing, yielding ≥10 Gb raw data per sample with Q30 ≥ 85%.

### 2.5. Co-Occurrence Network Construction and Analysis

The rhizosphere microbial co-occurrence network was constructed using genus-level relative abundance data from metagenomic sequencing. Genera present in ≥5 samples with relative abundance ≥0.02% were retained. Data were Hellinger-transformed via the vegan package in R v4.5.2, and significant pairwise Spearman correlations (|r| ≥ 0.7, *p* < 0.05) were identified using corrplot to generate an edge list [60]. The network was visualized in Gephi (version 0.10.1). Nodes (representing genera) were sized proportionally to their relative abundance. Positive and negative correlations were depicted by red and green edges, respectively. The layout was optimized using the Fruchterman Reingold algorithm with parameters set to 10,000 iterations, a gravity of 10, and a speed of 1.0 [61].

Network visualization was performed in Gephi v0.10.1. Nodes representing genera were sized by relative abundance. Positive and negative correlations were shown as red and green edges, while layout was optimized via the Fruchterman Reingold algorithm with 10,000 iterations, gravity = 10, and speed = 1.0. Topological properties, including nodes, edges, average degree, and modularity, were calculated in R using igraph and ggplot2. Nodes were classified into four topological roles based on Zi-Pi values [62] and color-coded by function, elucidating genus interactions, dominant taxa, and key roles under salt-alkali stress.

### 2.6. Partial Least Squares Path Modeling

To quantify microbial mediating roles, Partial Least Squares Path Modeling (PLS-PM) was implemented in R v4.5.2 using the plspm package. PLS-PM was conducted to elucidate cascading pathways in the soil–microbe–plant system under saline-alkali stress. Six latent variables were constructed based on measured indicators: soil physicochemical properties (pH, EC, SWC, TOC, TN), soil enzyme activities (S-UE, S-ALP, S-CAT, S-SC), microbial community (diversity indices and key taxa abundances), microbial function (metabolic pathway abundances and resistance gene abundances) [63], plant physiology (antioxidant enzyme activities, osmolytes, chlorophyll parameters), and plant growth (plant height increment, stem diameter increment). Path relationships were hypothesized based on a conceptual model in which soil properties drive microbial communities, which in turn influence soil enzyme activities and ultimately plant physiology and growth. The measurement model was evaluated using indicator loadings (>0.7), average variance extracted (AVE > 0.5), and composite reliability (CR > 0.7) following recommended thresholds. Overall goodness-of-fit (GoF) was calculated as the square root of the mean R^2^ of endogenous latent variables, with GoF > 0.36 indicating satisfactory fit [64].

### 2.7. Statistical Analysis

All results were expressed as the mean ± standard deviation (±SD) of three independent experiments. Statistical analyses were performed using Origin 2024 (SR1), Microsoft Excel 2016, and other software, with a significance level set at *p* < 0.05. For plant growth, physiological, and soil indices, normality and homogeneity of variances were tested first, followed by analysis of variance (ANOVA) for intergroup differences. Duncan‘s new multiple range test was used for post hoc comparisons following ANOVA. Graphs were plotted using Graphpad Prism 10.1.2 and Origin 2024. Microbial community composition and diversity were analyzed via R packages vegan (α/β diversity indices) and DESeq (community differences), with *p*-values adjusted using the Benjamini–Hochberg procedure to control the false discovery rate. Genus-level boxplots were generated by Origin 2024. LEfSe analysis was conducted on the Novogene Cloud Platform (https://magic.novogene.com/customer/main#/homeNew, accessed date 22 February 2026), while Mantel tests and redundancy analysis (RDA) were performed using Chiplot (https://chiplot.online/, accessed date 22 February 2026) and Canoco 5 (https://www.canoco5.com/, accessed date 22 February 2026), respectively. For Mantel tests performed via Chiplot, no multiple comparison correction was applied, as each test examined an independent hypothesis and the number of tests was limited. Microbial functional analyses were completed using STAMP software (Windows ver. 2.1.3) and R packages clusterProfiler (4.6.0), rhierbaps (1.1.0), and plspm (0.4.9). The relative abundance of functional genes and antibiotic resistance genes (ARGs) was calculated from three biological replicates, with standard errors determined. Functional enrichment and differential metabolic pathway analyses were implemented via clusterProfiler and STAMP (*p* < 0.05). Spearman’s correlation coefficients assessed associations between metabolic pathways, plant physiological indices, and soil properties, while partial Mantel tests evaluated correlations between soil properties and ARG profiles. Graphs were generated using R packages ggplot2 (ver. 3.2.0) and pheatmap (1.0.12), with heatmaps produced by pheatmap. Circos-algorithm-based genomic circle maps showing functional gene distribution were plotted on the Novogene Cloud Platform, and PLS-PM models were constructed through plspm and validated by Bootstrap resampling (1000 iterations).

## 3. Results

### 3.1. Differential Responses of Quercus mongolica Growth Physiology and Rhizosphere Soil Properties to Salt-Alkali Stress

#### 3.1.1. Effects of Salt-Alkali Stress on Growth Characteristics of *Quercus mongolica* Seedlings

**Figure 1:**
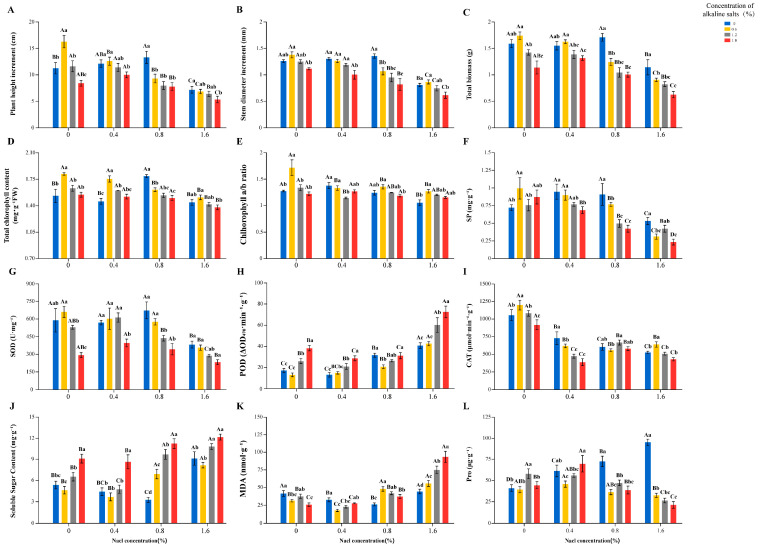
Effects of saline-alkali stress on the growth and physiological characteristics of *Quercus mongolica*. All values in the figure represent means ± standard deviation (*n* = 3). Different letters within the same column indicate significant differences between treatments at the 0.05 level. Lowercase letters denote differences between varying alkali concentrations at the same salt concentration; uppercase letters denote differences between varying salt concentrations at the same alkali concentration. (**A**) Plant height increment; (**B**) Stem diamter increment; (**C**) Total biomass; (**D**) Total chlorophyll content; (**E**) Chlorophyll a/b ratio; (**F**) SP: soluble protein; (**G**) SOD: superoxide dismutase; (**H**) POD: peroxidase; (**I**) CAT: catalase; (**J**) SS: soluble sugar; (**K**) MDA: malondialdehyde; (**L**) Pro: proline.

Under single salt stress, all three parameters were observed to attain maximum values at the S_2_ treatment (Figure 1, Table S1), with respective increases of 18.29%, 7.93%, and 7.55% relative to the CK, whereas their minimum values were recorded at the S_3_ treatment, corresponding to decreases of 36.94%, 35.71%, and 28.30%. A parallel trend was detected under single alkali stress (Figure 1). Maxima were attained at the A_1_ treatment with respective increases of 44.58%, 9.52%, and 9.43%, and minima were determined at the A_3_ treatment with respective decreases of 25.22%, 11.11%, and 28.30%. Growth performance was significantly suppressed under combined saline-alkali stress (Figure 1), with the S_3_A_3_ treatment inducing the most severe inhibition, corresponding to reductions of 52.58%, 50.79%, and 60.38% compared with the CK.

#### 3.1.2. Effects of Salt-Alkali Stress on Physiological Characteristics of *Quercus mongolica* Seedlings

Under single salt stress, SOD activity showed an overall decreasing trend (Figure 1, Table S1), with its maximum and minimum values recorded in the S_2_ and S_3_ treatment, respectively, representing a 14.27% increase and a 35.05% decrease compared to the CK, respectively. POD activity, MDA, and SS contents were found to decrease first and then increase with increasing salt concentration; the minimum POD activity was noted in the S_1_ group, while the minimum MDA and SS contents were both recorded in the S_2_ group (Figure 1). These three minimum values were 23.39%, 36.79%, and 38.69% lower than those in the CK, respectively, and their maximum values were all documented in the S_3_ group, representing a 136.10%, 6.89%, and 70.28% increase compared to the CK, respectively (Figure 1). An inverse variation trend was observed between CAT activity and Pro content, with a minimum of CAT activity noted in the S_3_ group, a 49.97% decrease compared to the CK, while Pro content was recorded at its maximum in the S_3_ group, a 131.89% increase relative to the CK. SP content showed a trend of increasing first and then decreasing, with its maximum value recorded in the S_1_ group, a 31.94% increase relative to the CK, and its minimum value noted in the S_3_ group, a 26.39% decrease relative to the CK (Figure 1).

Under single alkali stress, SOD and CAT activities were both observed as increasing first and then decreasing with increasing alkali concentration, with their maximum values recorded in the A_1_ group, a 12.06% and 13.51% increase compared to the CK respectively, and minimum values noted in the A_3_ group, a 50.10% and 12.96% decrease relative to the CK, respectively (Figure 1). POD activity and SS content showed a trend of decreasing first and then increasing, with their minimum values observed in the A_1_ group, representing a 24.03% and 13.64% decrease compared to the CK group, respectively, and maximum values noted in the A_3_ group, representing a 122.23% and 69.72% increase relative to the CK group, respectively (Figure 1). MDA and Pro contents exhibited an initial decrease, a subsequent increase, and a final decrease. The minimum values of MDA and Pro content were recorded in the A_3_ and A_1_ groups, showing decreases of 37.44% and 4.49%, respectively. The maximum Pro content value was recorded in the A_2_ group, representing a 41.21% increase relative to the CK (Figure 1). SP content showed an opposite trend to that of MDA and Pro, with its maximum value recorded in the A_1_ group, a 38.89% increase compared to the CK (Figure 1).

Under combined saline-alkali stress with a fixed salt concentration, SOD activity, SP content, and CAT activity were all found to show a decreasing trend with increasing alkali concentration, with their minimum values detected in the S_3_A_3_ and S_1_A_3_ treatment, corresponding to a 60.50%, 68.06%, and 63.17% decrease compared to the CK, respectively (Figure 1). In contrast, for POD activity and MDA, SS and Pro contents exhibited an increasing trend, with the maximum values of POD activity, MDA, and SS contents recorded in the S_3_A_3_, representing 321.13%, 124.85%, and 127.48% increases compared to the CK, respectively. The maximum Pro content was observed in the S_1_A_3_, representing a 69.81% increase relative to the CK (Figure 1).

Under single salt stress, the total chlorophyll content peaked at S_2_, with a significant increase of 16.99% compared to the CK. Conversely, the maximum of the chlorophyll a/b ratio was found in the S_1_ group, which was 7.81% higher than the CK (Figure 1). However, when the salt concentration was raised to S_3_, the total chlorophyll content decreased by 5.89%, and the chlorophyll a/b ratio decreased by 17.97%, relative to the CK (Figure 1). Under single alkali stress, peak values of total chlorophyll content and chlorophyll a/b ratio were shown in the A_1_ groups, increasing by 18.95% and 34.38% relative to the CK, respectively (Figure 1). With increasing alkali concentration, total chlorophyll remained marginally above the CK at A_3_, whereas the chlorophyll a/b ratio was decreased to its minimum, showing a 4.68% decrease (Figure 1). Under combined saline-alkali stress, total chlorophyll content exhibited an overall decreasing trend, whereas no significant change was observed in the chlorophyll a/b ratio (Figure 1). However, at the same salt concentration, parameters declined with increasing alkali levels. The S_3_A_3_ treatment could be considered a threshold for stress effects. In this treatment, total chlorophyll content and the chlorophyll a/b ratio were slightly elevated compared to the CK, by 5.23% and 6.25%, respectively (Figure 1).

#### 3.1.3. Effects of Salt-Alkali Stress on Soil Characteristics in the Rhizosphere of *Quercus mongolica* Seedlings

**Figure 2:**
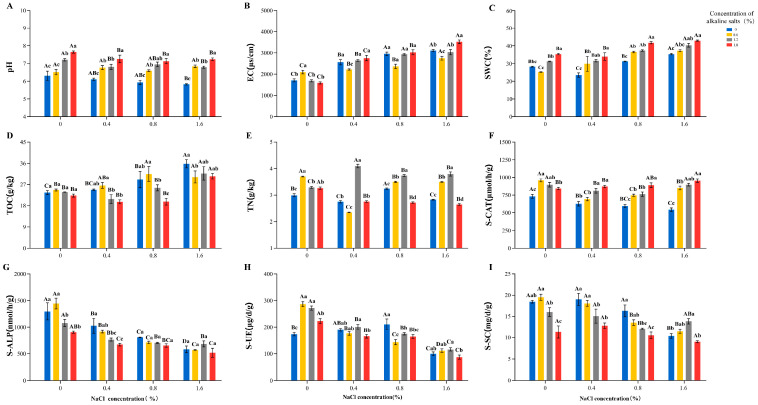
Effects of saline-alkali stress on the physicochemical properties of *Quercus mongolica* rhizosphere soil. All values in the figure represent means ± standard deviation (*n* = 3). Different letters within the same column indicate significant differences between treatments at the 0.05 level. Lowercase letters denote differences between varying alkali concentrations at the same salt concentration; uppercase letters denote differences between varying salt concentrations at the same alkali concentration. (**A**) pH; (**B**) EC: electrical conductivity; (**C**) SWC: soil water content; (**D**) TOC: total organic carbon; (**E**) TN: total nitrogen; (**F**) S-CAT: soil catalase; (**G**) S-ALP: soil alkaline phosphatase; (**H**) S-UE: soil urease; (**I**) S-SC: soil sucra.

Multiple rhizosphere soil indicators of *Q. mongolica* seedlings were analyzed, and distinct indicator-specific response patterns of soil physicochemical properties were induced by different stress types (Figure 2, Table S2). Under single salt stress, pH, S-CAT, and S-ALP were found to decrease with rising salt concentration, with minima recorded at S_3_, which were reduced by 7.61%, 30.55%, and 37.87% relative to the CK, respectively. Conversely, EC, SWC, and TOC underwent a gradual increase to maxima at S_3_, with respective increases of 82.21%, 43.70%, and 27.90% in comparison with the CK, respectively. An initial increase followed by a decrease was exhibited by S-UE and S-SC. The maximum of S-UE was attained at S_2,_ corresponding to an increase of 21.52% relative to the CK. The maximum of S-SC was achieved at S_1_ with an increase of 2.92% compared with the CK. The minimums of both indicators were recorded at S_3_, which were reduced by 42.28% and 43.81% in comparison with the CK, respectively.

Under single alkali stress, pH was observed to increase steadily with rising alkali concentration, with the maximum attained at A_3_ and a 21.24% increase relative to the CK. EC, TOC, TN, S-CAT, S-ALP, S-UE, and S-SC were found to reach their maximum at the A_1_, exceeding the CK by 22.89%, 4.76%, 23.75%, 30.83%, 11.41%, 65.50% and 5.68%, respectively. At A_3_, TN, S-CAT, and S-UE were detected to remain above the CK, though reduced from their peak values at A_1_, while EC, TOC, S-ALP, and S-SC were observed to decline to their minimum values, which were 6.84%, 5.27%, 30.43%, and 38.67% lower compared with the CK. SWC exhibited a unique response pattern, which decreased by 10.71% at A_1_ relative to the CK and subsequently rose to its peak at A_3_ with a 25% increase in comparison with the CK.

Under combined saline-alkali stress with a constant salt concentration, distinct differences were observed in how various soil properties responded to increasing alkali levels. Soil pH, EC, SWC, and S-CAT were demonstrated to increase steadily as the alkali concentration increased progressively, with their maximum values all recorded in S_3_A_3_. These parameters were increased by 14.74%, 106.45%, 53.57%, and 29.96%, respectively, compared with the CK. In contrast, TN and S-UE activity exhibited fluctuating changes, with a decrease–increase–decrease trend identified, with the alkali concentration elevated continuously, and their lowest values were detected in S_3_A_3_, which were reduced by 11.37% and 49.46%, respectively. TOC, S-ALP, and S-SC activity showed an overall decreasing trend as alkali concentration increased.

### 3.2. Variations in Rhizosphere Soil Microbial Community Composition and Diversity

To clarify the microbial role in promoting *Q. mongolica* seedling growth under low-concentration single salt or alkali stress and its inhibitory role under combined stress, metagenomic sequencing was employed in this study to analyze rhizosphere soil microbial communities from four groups with three biological replicates each, including the CK, S_2_, A_1_, and S_2_A_1_. Raw sequencing data of 137,540 Mbp were generated, with 198 phylum-level and 4228 genus-level taxa identified. Genus-level taxonomic composition remained stable across treatments, whereas significant differences in the relative abundance were detected.

#### 3.2.1. Analysis of Community Composition and Diversity

**Figure 3:**
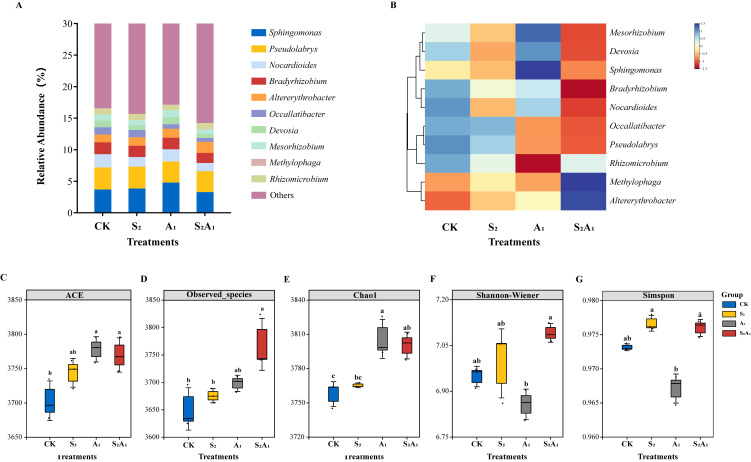
Soil microbial composition and α diversity indices in the rhizosphere of *Quercus mongolica* under saline-alkali stress. (**A**) Microbial genus-level community composition map; (**B**) genus-level clustering heatmap. Genus-level α-diversity indices: (**C**) ACE index; (**D**) Observed_species index; (**E**) Chao1 index; (**F**) Shannon–Wiener index; (**G**) Simpson index for four rhizosphere soil samples. All values in the table represent means ± standard deviation (*n* = 3). Lowercase letters indicate significant differences among the four samples (*p* < 0.05, Tukey’s HSD post hoc analysis).

At the genus level, *Sphingomonas*, *Pseudolabrys*, *Nocardioides*, *Bradyrhizobium*, and *Altererythrobacter* were identified as dominant genera. *Occallatibacter*, *Devosia*, and *Mesorhizobium* were determined as secondary dominant genera (Figure 3A,B and Table S3). Under the S_2_ treatment, no significant overall difference from the CK was noted, but *Sphingomonas*, *Nocardioides*, and *Devosia* exhibited slight decreases. *Altererythrobacter* and *Pseudomonas* showed marginal increases. Under the A_1_ treatment, the abundances of *Sphingomonas*, *Devosia,* and *Mesorhizobium* increased, whereas *Pseudolabrys*, *Occallatibacter,* and *Rhizomicrobium* decreased. More pronounced shifts were detected under the S_2_A_1_ treatment, with the abundances of most genera decreasing significantly; exceptions were *Altererythrobacter* and *Mesorhizobium*, whose abundances continued to increase.

Significant inter-group variations were exhibited by rhizosphere soil microbial richness and diversity indices across four treatments (Figure 3). Among richness indices, minimum ACE and Chao1 index values were assigned to the CK, while peak values were attributed to A_1_ (Figure 3C,D). The minimum observed_species number value was also recorded in the CK, with its maximum assigned to S_2_A_1_ (Figure 3E). In contrast, the extreme-value attribution pattern of diversity indices differed from that of richness indices. The minimum Shannon–Wiener index value was documented in A_1_ and its maximum attributed to S_2_A_1_, while Simpson’s minimum was consistent with Shannon’s, and its peak was assigned to S_2_ (Figure 3F,G).

#### 3.2.2. Linear Discriminant Analysis

**Figure 4:**
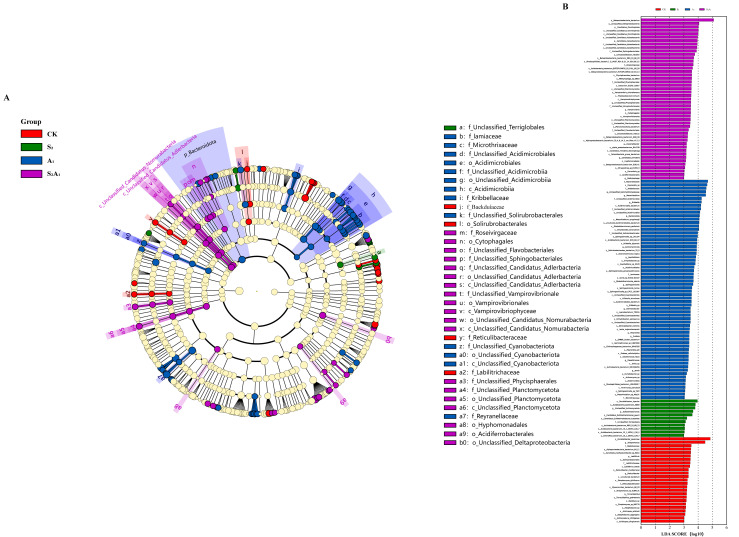
Analysis of microbial species differences in the rhizosphere of *Quercus mongolica* under different treatments. (**A**) Phylogenetic tree; (**B**) distribution map, showing the number of different microorganisms at each taxonomic level under different treatments.

Linear discriminant analysis effect size (LEfSe) was employed, with a threshold value of 3 set as the screening criterion, to identify statistically significant differential biomarkers across treatment groups. A total of 37 differential biomarkers were identified (Figure 4B). Among these biomarkers, *Beakduiaceae*, *Reticulibacteraceae,* and *Labilitrichacea* were defined as the characteristic differential biomarkers of the CK (Figure 4A). *Unclassified_Terriglobales* was confirmed as the core differential species in the S_2_ treatment (Figure 4A). The number of differential biomarkers in the A_1_ treatment was higher than that in both the CK and S_2_ treatments, with *Bacteroidota* and *Acidimicrobiia* showing the most significant differences (Figure 4A). Under the S_2_A_1_ treatment, *Unclassified_Candidatus_Nomurabacteria*, and *Unclassified_Candidatus_Adlerbacteria* were identified as the characteristic differential biomarkers (Figure 4A). The effect sizes for all treatment groups were above 3, with some exceeding 4.

### 3.3. Association Analysis of Plant Physiology, Rhizosphere Soil Properties, and Microbial Community

**Figure 5:**
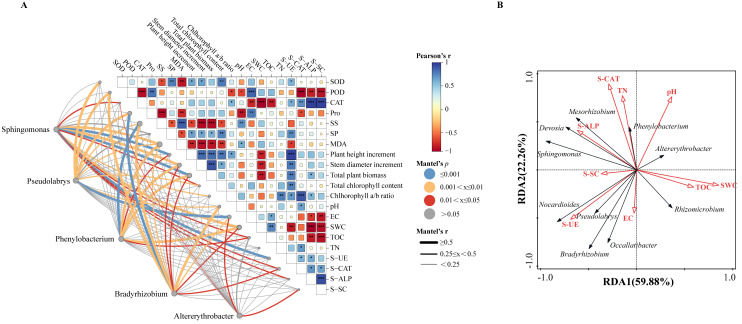
Relationship between physiological parameters and soil physicochemical properties of *Quercus mongolica* under different treatments and rhizosphere microorganisms. (**A**) Mantel correlation analysis between physiological indicators, soil physicochemical properties, and the five most abundant genera of rhizosphere microbial community structure (*p* < 0.05). Color shading indicates variations in Pearson correlation coefficients. The size of the squares represents the significance level (larger squares indicate greater significance), while the color of the squares indicates the direction (positive or negative) and magnitude of the correlations. The circles represent the significance of associations between microorganisms and various indicators, with circle size reflecting the significance level. Asterisks denote statistical significance: * at *p* < 0.05; ** at *p* < 0.01; *** at *p* < 0.001 (**B**) RDA of rhizosphere microbial relative abundance at the genus level and soil physicochemical properties. The red lines represent environmental factors and the black lines represent microorganisms.

To identify microbial factors associated with physiological and soil physicochemical indices linked to *Q. mongolica* seedling growth, a Mantel test was performed on 21 indices. Different correlations between genus-level microbial communities and these indices across treatments were observed (Figure 5A). Pearson correlation analysis was conducted. CAT showed strong positive correlations with S-ALP and S-SC. Plant height increment was confirmed to show positive correlations with stem diameter increment, total biomass, and S-UE. Conversely, POD was identified as negatively correlated with CAT, S-AT, S-ALP, and S-SC. SS was determined to be inversely correlated with stem diameter. Total biomass and chlorophyll. EC, SWC, and TOC exhibited strong negative correlations with S-ALP and S-SC. Correlation analysis of five dominant genera revealed that the prominent associations through thicker blue and orange-red lines (*p* < 0.01) were represented by *Sphingomonas* and *Pseudomonas*, while weaker associations through thinner gray lines (*p* > 0.05) were demonstrated by *Bradyrhizobium*.

RDA was employed to reveal the correlations between genus-level rhizosphere soil microorganisms and soil physicochemical properties. 59.88% and 22.26% of the variation in microbial community structure were accounted for by the first and second ordination axes, respectively (Figure 5B). The results showed that the arrow lengths of SWC, S-UE, and S-CAT were the longest. *Sphingomonas*, *Devosia*, *Mesorhizobium,* and *Phenylobacterium* exhibited positive correlations with S-ALP, S-CAT, and TN. The pH was shown to be closely correlated with the abundance distribution of *Altererythrobacter*. Meanwhile, genera including *Bradyrhizobium* and *Pseudolabrys* were characterized by positive correlations with S-UE, S-SC, and EC, whereas positive correlations with TOC and SWC were demonstrated by genera such as *Rhizomicrobium*.

### 3.4. Characterization of Microbial Co-Occurrence Network

**Figure 6:**
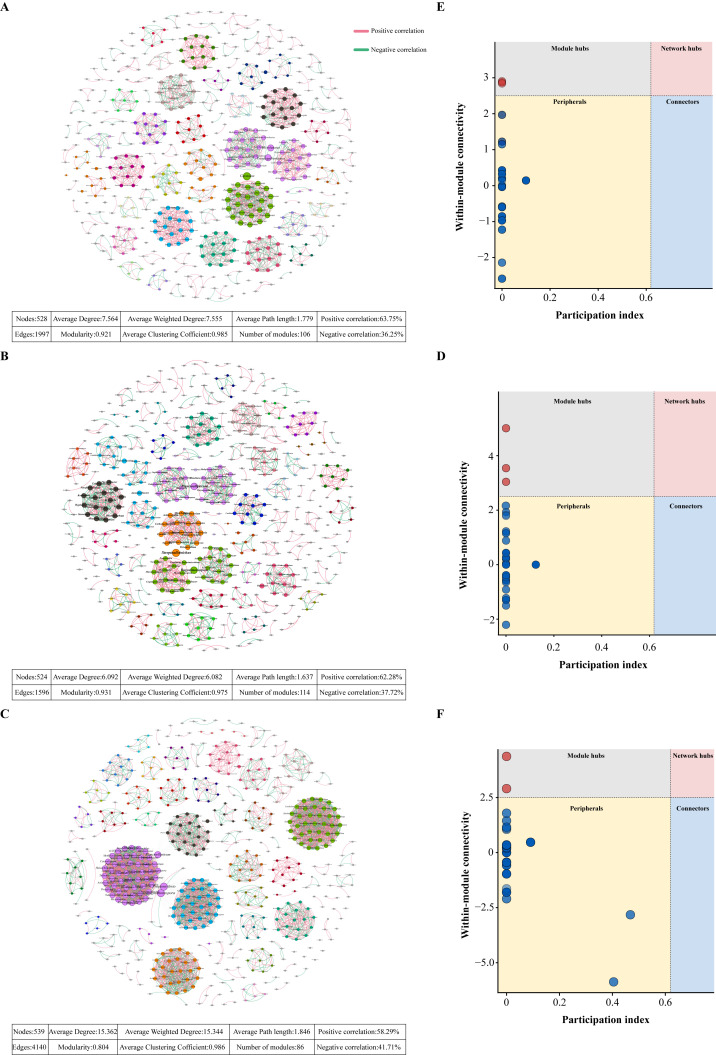
Topological structure and module property analysis of rhizosphere soil microbial co-association network. (**A**,**C**,**E**) show the topological structures of microbial co-occurrence networks between CK and salt stress (S_2_), CK and alkali stress (A_1_), and CK and saline-alkali composite stress (S_2_A_1_), respectively. Each node represents a microbial genus. Node size is proportional to the relative abundance of the genus. Node colors indicate different modules identified by the network analysis. Edges represent significant correlations based on Spearman’s rank correlation coefficient (|r| > 0.7, *p* < 0.05). Edge colors denote positive (red) and negative (green) correlations. (**B**,**D**,**F**) present the scatter plots of within-module connectivity contrast to participation index corresponding to the above stress comparisons, respectively, where red nodes represent Module hubs and blue nodes represent Peripherals.

The structure of the rhizosphere soil microbial co-occurrence network of *Q. mongolica* seedlings was significantly influenced by saline-alkali stress. In the CK versus S_2_ comparison group, the number of nodes, edges, average degree, average weighted degree, and modularity coefficient of the network were all intermediate between the corresponding indices of the other comparison networks. The proportion of positive correlations among microorganisms reached the maximum proportion of 63.75% (Figure 6A). Three module hub species, namely *Pseudogulbenkiania*, *Microlunatus*, and *Azoarcus*, were identified from the co-occurrence network through Zi-Pi topological analysis (Figure 6B).

In the CK versus A_1_ comparison group, the number of nodes, edges, average degree, average weighted degree, and average path length of the network were the lowest among the three groups, whereas its modularity coefficient and number of modules were found to attain the maximum values of 0.931 and 114, respectively (Figure 6C). *Aurantiacibacter* and *Rugosimonospora* were identified as the module hub species in this network (Figure 6D).

In the CK versus S_2_A_1_ comparison group, the number of nodes, edges, average degree, average weighted degree, and average clustering coefficient of the co-occurrence network were higher than those of the other two groups. However, compared with the networks, this group showed the lowest modularity coefficient, number of modules, and proportion of positive correlations among microorganisms, the latter of which decreased to 58.29% (Figure 6E). Two module hub species that met the screening criteria (Zi < 2.5 and Pi < 0.62) were identified through Zi-Pi topological analysis, namely Aurantiacibacter and Thermogemmata (Figure 6F).

### 3.5. Effects of Salt-Alkali Stress on the Functional Potential of Microbial Communities

#### 3.5.1. Response of Core Metabolic Pathways Based on the KEGG Database

**Figure 7:**
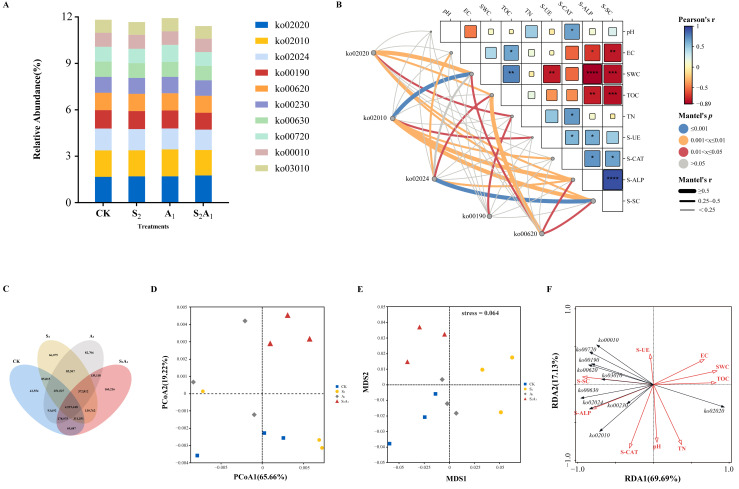
Multidimensional analysis of soil microbial KEGG pathway abundances, environmental factor associations, and community structure under different treatments. (**A**) Stacked bar plot of the relative abundances of KEGG functional pathways. Different colors represent distinct functional pathways. (**B**) Mantel correlation heatmap between KEGG pathways and environmental factors. In the heatmap, squares represent correlations among soil physicochemical properties: the size of each square reflects the significance level (larger squares indicate more significant correlations), while the color indicates the direction (positive/negative) and magnitude of the correlation. The color and thickness of lines represent the significance and strength of the associations between functional pathways and physicochemical properties. Significance levels are denoted by asterisks: * *p* < 0.05, ** *p* < 0.01, *** *p* < 0.001, **** *p* < 0.0001. (**C**) Venn diagram showing the number of genes assigned to KEGG pathways. (**D**) PCoA plot of KEGG functional pathways. (**E**) NMDS plot of KEGG functional pathways. The value at the top-right corner of the plot is the stress value. (**F**) RDA plot between KEGG pathways and environmental factors. Black arrows represent various soil physicochemical properties, and red arrows represent distinct KEGG functional pathways.

To clarify the functional traits closely associated with microbial populations, KEGG functional annotation was conducted (Figure 7). It was observed that among the top 10 third-level functional pathways sorted by relative abundance, the overall inter-group differences in expression levels were minor, with only a few pathways exhibiting significant inter-group differentiation (Figure 7A). It was determined that under the S_2_, only the abundance of ko02020 was higher than the CK. Under A_1_, the abundances of ko02020 and ko01010 were identified to be distinctly elevated relative to the CK (Figure 7A). Compared with the other three groups, the S_2_A_1_ group exhibited the most significant increase in the abundance of ko02020, while the other pathways showed a decreasing trend (Figure 5A). Mantel analysis was performed, showing that ko02020 was closely correlated with SWC, TOC, S-CAT, S-ALP, and S-SC. ko02010 exhibited the strongest association with SWC, followed by that with S-ALP and S-SC. ko02024 was tightly linked to S-SC variations. ko00190 correlated exclusively with TOC content, and ko00620 abundance changes were shown to be associated with TOC, SWC, S-ALP, and S-SC (Figure 7B). A Venn diagram of gene counts was generated, and it was revealed that the combined saline-alkali stress group shared the largest associated gene number with the other three groups (Figure 7C). Principal coordinate analysis (PCoA) was carried out, and it was illustrated that axes 1 and 2 explained 65.66% and 19.22% of the total sample variation, respectively. The combined saline-alkali stress group exhibited distinct separation from other groups (Figure 7D). Non-metric multidimensional scaling (NMDS) was implemented, and a stress value of 0.064 was obtained. This value indicated satisfactory fitting consistency with PCoA results (Figure 7E). RDA showed that environmental factors collectively explained 86.82% of the variation in microbial KEGG pathway abundance, with S-SC, TOC, and S-CAT identified as the most significant regulatory drivers (Figure 7F). Correlation analysis was conducted, and it was found that ko00010 and ko00720 were positively correlated with S-SC, ko00630 was negatively correlated with TN, and ko00910 was positively correlated with TN (Figure 7F).

#### 3.5.2. Responses of Antibiotic Resistance Genes Based on the CARD

**Figure 8:**
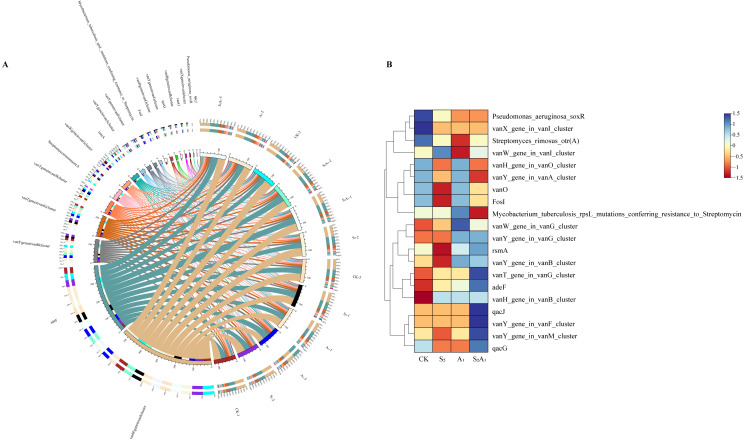
CIRCOS plot and clustering heatmap of the type, abundance, and classification of CARD-annotated antibiotic resistance genes in rhizosphere soil microorganisms under different treatments. (**A**) CIRCOS plot of rhizosphere soil microorganisms under different treatments. The colored bands on the left side of the inner ring, corresponding to the right side of the outer ring, represent different resistance genes, with chords indicating associations between genes and samples. The colored modules on the right side of the inner ring, corresponding to the left side of the outer ring, represent different samples. The left side of the outer ring shows the abundance of each resistance gene across samples, while the right side shows the abundance of each resistance gene within samples. (**B**) Clustering heatmap of the top 20 resistance genes in terms of abundance under different treatments. Different colors indicate the direction (positive/negative) and magnitude of correlations.

Non-redundant gene sets were annotated against the resistance gene database and subsequently compared with the Comprehensive Antibiotic Resistance Database (CARD). The top 20 antibiotic resistance genes in the CARD from *Q. mongolica* rhizosphere soil samples under different treatments were compared (Figure 8A). Results showed that antibiotic resistance gene expression levels were most significant under A_1_ and S_2_A_1_, with notable differences with the CK (Figure 8B). Only the *vanW_gene_in_vanl_cluster gene* showed a significant positive correlation under S_2_, while the overall expression level of resistance genes remained low (Figure 8B). Under A_1_, *vanW_gene_in_vanG_cluster* and other genes, including *rsmA* and *adeF*, exhibited a significant divergence from the CK (Figure 8B). Under S_2_A_1_, genes including *qacJ* were confirmed to present distinct variation relative to the CK, with their expression levels increasing substantially (Figure 8B).

### 3.6. Partial Least Squares Path Modeling (PLS-PM) Analysis

**Figure 9:**
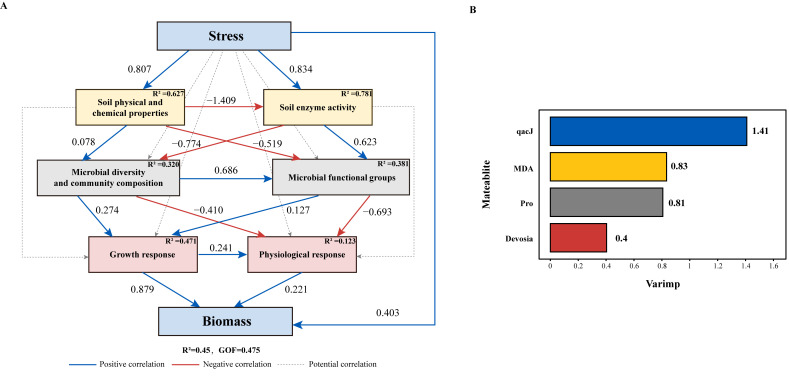
PLS-PM path model and effect importance plot under different saline-alkali stress treatments. (**A**) PLS-PM path model diagram of soil and microbial characteristics, growth, and physiology under different saline-alkali stresses. Blue boxes represent independent and dependent variables; yellow, gray, and red borders denote latent variables at different levels. Blue arrows indicate positive correlations, red arrows indicate negative correlations, and gray arrows represent potential correlations. The numbers next to the arrows are the path coefficients between latent variables, R^2^ represents the mean value, and GOF represents the goodness of fit of the entire model. (**B**) Effect importance plot corresponding to the PLS-PM model. The horizontal axis represents varimp values, and the vertical axis represents different observed variables.

It was indicated by PLS-PM analysis that saline-alkali stress at the first level induced a strong positive effect on soil physicochemical properties and enzyme activities (Figure 9A). At the second level, soil physicochemical properties were observed to have a strong positive effect on microbial diversity and composition. However, they showed a strong negative correlation with microbial functional groups. In contrast, soil enzyme activities exhibited the opposite trend (Figure 9A). At the third level, microbial diversity was found to mildly promote plant growth. However, it was also found to inhibit physiological responses. Microbial functional groups were revealed to produce a weaker effect. Additionally, a significant negative trade-off was identified between plant growth and physiological responses (Figure 9A). Plant growth was defined as the core biomass driver; saline-alkali stress was confirmed to generate a direct, strong promotion effect on biomass (Figure 9A).

The evaluation of the PLS-PM measurement model showed that all latent variables except microbial function (Func) met the recommended thresholds for indicator loadings (>0.7), average variance extracted (AVE > 0.5), and composite reliability (CR > 0.7), indicating generally good convergent validity and internal consistency. The Func latent variable had slightly lower AVE (0.41) and CR (0.43), likely reflecting the distinct functional dimensions captured by metabolic pathway and resistance gene abundances (Table S4). The model’s GoF value was determined to be 0.475 (above 0.36 threshold), confirming a good fit. Its mean R^2^ was relatively small but still explained 45% of biomass variation. The varimp value of *qacJ* was measured to reach 1.41, recognized as the top core variable, followed by MDA, Pro, and *Devosia* (Figure 9B).

## 4. Discussion

As a woody glycophyte typically considered salt-sensitive, *Q. mongolica* unexpectedly exhibited growth promotion under low-concentration salt stress (100 mmol·L^−1^ NaCl) and alkaline stress (50 mmol·L^−1^ Na_2_CO_3_), displaying a clear biphasic hormetic response. This deviates from the expected pattern of monotonic growth reduction with increasing salinity commonly observed in glycophytes [65,66]. This unusual behavior suggests that the boundary between glycophytic and halophytic strategies may not be absolute for woody perennials [67], which can exhibit intermediate adaptive responses depending on stress intensity and duration. The following sections discuss the underlying mechanisms for this hormetic response under each stress type as well as the contrasting inhibitory effects under combined stress.

### 4.1. The Growth-Promoting Effects and Underlying Mechanisms of Low-Concentration Salt Stress

Extensive research indicates that sublethal salt stress can serve as an ecological signal to induce adaptive responses in plants, manifesting as a hormetic effect that stimulates growth at low concentrations [68,69]. This study reveals further evidence that *Q. mongolica* seedlings exhibit a biphasic dose-response pattern to salt stress, characterized by growth promotion at low concentrations and inhibition at high concentrations.

#### 4.1.1. Soil Physicochemical Changes Under Salt Stress

Under salt stress, the rhizosphere microenvironment exhibits a typical ion-dominated response pattern. This pattern manifests through three interconnected aspects. First, the increased influx of Na^+^ elevates rhizosphere EC, which in turn activates the root plasma membrane H^+^-ATPase to extrude H^+^. This process achieves Na^+^ exclusion and induces rhizosphere acidification [70,71]. This root-mediated acidification is regarded as a fundamental mechanism for enhancing plant salt tolerance [28]. Second, salt ions decrease soil water potential, triggering physiological water retention, as evidenced by increased SWC (Figure 2). Consequently, a decrease in osmotic potential reduces plant-available water, leading to physiological drought [72,73]. Third, the synchronous accumulation of TOC and TN (Figure 2) may reflect alterations in carbon and nitrogen cycling processes. This pattern is consistent with the inhibitory effects of salt ions on microbial-mediated turnover of organic matter, as previously reported [74].

#### 4.1.2. Microbial Community and Functional Responses Under Salt Stress

This selective pressure is directly associated with a multi-level, systematic restructuring of microbial community [75], selecting for and enriching taxa with specific adaptive strategies, such as *Pseudolays* [76]. This genus likely contributes to host homeostasis under salt stress through three complementary mechanisms: ACC deaminase production reduces stress-induced ethylene levels, preventing premature senescence; exopolysaccharide secretion enhances soil aggregation and binds Na^+^ ions, reducing their availability to plants; and ion transport regulation may facilitate K^+^/Na^+^ homeostasis in the rhizosphere [77,78]. These microbe-mediated processes collectively alleviate ion toxicity and oxidative stress, explaining the observed enhancement of plant antioxidant capacity. Concurrently, root-secreted organic acids restructure microbial networks through rhizosphere modulation, thereby enhancing systemic stress resistance by fostering positive interactions and functional synergies within these networks [79]. Hub species such as *Pseudogulbenkiania* and *Microlunatus* (Figure 2) were identified as potential keystone taxa associated with network stability [80]. Functional gene analysis revealed two key adaptive strategies at the molecular level: increased abundance of the ko02020 two-component system pathway indicates enhanced microbial capacity for sensing and responding to osmotic and ionic signals; enrichment of the *vanW* resistance gene reflects genetic-level adaptation to cell wall stress under high salinity. Together, these adjustments enhance community-level salt tolerance [81,82], maintaining a functional microbiome capable of supporting plant growth under stress. By inhibiting overall microbial function and thereby causing a decreased rate of organic matter decomposition, the restructuring of the microbial community is associated with a general decline in soil enzyme activity [83,84]. An exception was observed for S-UE, whose activity increased relative to the CK (Figure 2), presumably as an adaptive response to nitrogen limitation through enhanced urea hydrolysis [85].

#### 4.1.3. Plant Physiological Responses Under Salt Stress

Synergistic regulation of physicochemical, microbial, and enzymatic activities ultimately impacted the physiology of *Q. mongolica* seedlings. Characteristic activation of the antioxidant enzyme system was observed, with significantly increased SOD and POD activities (Figure 1). This response corresponds to the primary oxidative damage pathways induced by salt stress [86]: enhanced SOD activity converts superoxide radicals into H_2_O_2_, while increased POD activity subsequently detoxifies H_2_O_2_, maintaining redox homeostasis and regulating ROS within a controlled range. This coordinated antioxidant defense alleviates membrane lipid peroxidation, as evidenced by reduced MDA content [87,88,89]. A significant accumulation of Pro was observed (Figure 1), providing osmotic adjustment [90] by stabilizing protein structures and maintaining cellular turgor under reduced water potential. In addition, an increase in total chlorophyll content, alongside a stable chlorophyll a/b ratio, was observed in seedlings (Figure 1), indicating structural integrity of photosystem II (PSII) reaction centers and light-harvesting complexes (LHCs). This preservation of photosynthetic apparatus, supported by microbial-mediated nutrient availability and reduced oxidative damage, enables optimized light-energy utilization efficiency [91].

Collectively, these rhizosphere-to-plant responses constitute a coordinated hormetic mechanism. At low salt concentrations, moderate selective pressure activates rhizosphere processes and plant defense systems with minimal energy cost, yielding net benefits such as maintained photosynthetic capacity and membrane integrity. This growth promotion is underpinned by enhanced antioxidant and osmotic regulation (e.g., increased SOD/POD activities, Pro accumulation), supported by rhizosphere microbial activities such as *Pseudolays* enrichment. This multi-level coherence provides empirical evidence for the hormesis threshold in a woody species and underscores the energetic trade-off underlying plant stress adaptation. These findings also suggest that moderate salt regimes could promote seedling establishment in saline habitats, while excessive accumulation risks disrupting microbe-mediated support systems.

### 4.2. The Growth-Promoting Effects and Underlying Mechanisms of Low-Concentration Alkali Stress

A similar biphasic response is observed in *Q. mongolica* seedlings under both alkaline and salt stress, characterized by growth promotion at low concentrations and inhibition at high concentrations. However, the optimal concentration range for growth promotion differs between the two stressors.

#### 4.2.1. Soil Physicochemical Changes Under Alkaline Stress

Under low-concentration alkaline stress, a distinct set of interconnected changes occurs in the rhizosphere environment. First, increases in pH, EC, and TN were observed (Figure 2). The increase in TN content may be related to the suppression of ammonia volatilization under alkaline conditions [92]. Second, the activities of soil enzymes, including S-UE, S-ALP, S-CAT, and S-SC, were synergistically enhanced (Figure 2). Mechanistically, the increase in S-UE and S-ALP activities is attributed to the elevated pH, which optimizes their catalytic microenvironment, as both enzymes exhibit optimal activity in neutral to alkaline conditions [93]. In parallel, the increase in S-CAT and S-SC may be associated with the enrichment of alkaline-tolerant microorganisms and accelerated carbon turnover [94]. Together, these coordinated responses contribute to a positive feedback loop between alkaline stress and soil enzyme activity [95], which alleviates stress-induced nutrient limitations. As a direct consequence of these physicochemical and enzymatic shifts, the rhizosphere microbial community undergoes multi-level functional restructuring in response to elevated pH [96].

#### 4.2.2. Microbial Community and Functional Responses Under Alkaline Stress

At the population level, taxa such as *Sphingomonas*, *Devosia*, and *Mesorhizobium* were enriched (Figure 4), which are recognized for their dual roles in plant growth promotion and stress tolerance [97]. This genus likely improves plant performance through multiple mechanisms that are particularly beneficial under alkaline conditions: phytohormone secretion (e.g., IAA) can directly stimulate root growth; phosphate solubilization increases P availability, which is often limited in alkaline soils; and enhanced nitrogen transformation may alleviate N limitation induced by high pH [98,99,100]. Furthermore, co-occurrence network analysis revealed a significant increase in community modularity, indicating the formation of more specialized and functionally cohesive microbial groups adapted to the alkaline environment [101]. Within this restructured network, hub species, including *Aurantiacibacter* and *Rugosimonospora,* were identified as potential keystone taxa associated with module stability [97]. Functional gene analysis provided further insight into microbial adaptation strategies. The increased abundances of the ko02020 two-component system and ko01010 energy metabolism pathways (Figure 5) may reflect enhanced microbial capacity for environmental signal perception and nutrient acquisition in response to alkaline conditions [15,102]. Additionally, the activation of resistance genes, such as *rsmA*, *vanY*, and *adeF* (Figure 6), indicates that multiple genetic-level defense mechanisms—including stress response regulation (*rsmA*), cell wall modification (*vanY*), and multidrug efflux (*adeF*)—were recruited to enhance microbial survival under alkaline conditions [103,104].

#### 4.2.3. Plant Physiological Responses Under Alkaline Stress

These adaptations collectively enhance the adaptability of the rhizosphere microbiome, which in turn enables it to support plants under stress. The enrichment of growth-promoting taxa, restructuring of microbial networks, and activation of stress-adaptive functional genes created a favorable rhizosphere environment that facilitated plant physiological adaptation. This was evidenced by enhanced antioxidant capacity (increased SOD and CAT activities with reduced MDA content, Figure 1), indicating efficient ROS scavenging and protection against oxidative membrane damage [105]. In contrast to salt stress, seedlings under alkaline stress preferentially accumulated SP (Figure 1), reflecting a metabolic shift toward synthesis of functional proteins (e.g., stress-responsive enzymes, chaperones) that support cellular function under high pH [42,92,106]. Concurrently, increases in both total chlorophyll content and the chlorophyll a/b ratio were observed (Figure 1), suggesting maintained PSII integrity and light-harvesting efficiency, indicating that photosynthetic machinery was preserved under alkaline stress.

In summary, the adaptive growth of *Q. mongolica* under low-concentration alkaline stress is supported by a multi-tiered network spanning the rhizosphere environment, microbial function, soil enzymes, and plant physiology. This integrated response constitutes a coordinated hormetic mechanism distinct from salt stress, characterized by pH-driven optimization of enzyme catalysis, enrichment of alkaline-tolerant taxa (e.g., *Sphingomonas*), and enhanced community modularity. These rhizosphere adjustments occur with minimal energy cost yet yield improved nutrient availability, maintained photosynthetic capacity, and enhanced antioxidant protection. The metabolic shift toward soluble protein accumulation rather than osmotic regulators reflects a stressor-specific adaptive strategy, providing empirical evidence for species-specific hormesis thresholds and energetic trade-offs shaping adaptation to different stress types. These findings also suggest that moderate alkalinity could be utilized to enhance seedling performance in alkaline soils, while highlighting soluble protein as a potential indicator for assessing plant adaptive status under alkaline conditions.

### 4.3. The Inhibitory Effects and Underlying Mechanisms of Combined Salt-Alkali Stress

The synergistic inhibitory effect of saline-alkali combined stress on *Q. mongolica* seedlings is mediated by a cascading pathway from the rhizosphere to the plant.

#### 4.3.1. Soil Physicochemical Changes Under Combined Stress

This synergistic pathway initially induces a severe deterioration of the rhizosphere soil environment through multiple interacting mechanisms. First, the combined stress significantly increases EC and SWC (Figure 2), reflecting enhanced osmotic stress and ion toxicity that directly impair microbial cell stability [107,108]. Second, the elevated TOC and TN contents were observed (Figure 2). Mechanistically, these increases likely result from two concurrent processes: the suppression of microbial decomposition and nitrogen fixation functions under stress, coupled with a relatively increased input of root exudates [109].

#### 4.3.2. Microbial Community and Functional Responses Under Combined Stress

This degraded soil environment disrupted microbial community structure and function. Unlike under single stressors, networks under combined stress exhibited high complexity but low synergy (Figure 4). This topological shift indicates that cooperative metabolic exchanges (e.g., cross-feeding) were replaced by competitive interactions, impairing community functional integration [59]. Within this loosened network, keystone taxa, such as *Aurantiacibacter* and *Thermogemmatispora,* were associated with reduced stability, suggesting that these taxa were unable to coordinate cross-module functions essential for community resilience under dual stress [110]. Only a limited number of taxa, including *Altererythrobacter* and *Mesorhizobium*, were significantly enriched. *Altererythrobacter* is primarily associated with soil electrical conductivity and salinity [111]. Although *Mesorhizobium* is recognized for its plant growth-promoting potential, including nitrogen fixation, this capacity is compromised under high salinity and pH conditions, which significantly suppress nodulation and nitrogen fixation efficiency [112]. Functional gene analysis revealed a metabolic reprioritization from mutualistic functions toward stress adaptation and cellular maintenance, evidenced by the suppression of core metabolic pathways alongside the activation of ko02020 (Figure 5) and the resistance genes *qacJ* and *vanT* (Figure 6). The ko02020 two-component system enhances bacterial signal perception for stress survival, while resistance gene activation reflects genetic-level adaptation to oxidative stress and membrane damage [113]. Together, these shifts indicate that microbial energy allocation was redirected from growth-promoting functions (e.g., nutrient cycling) toward self-preservation. The functional imbalance of the microbial community was reflected in differential responses in soil enzyme activities. Notably, elevated S-CAT activity may indicate an intense microbial antioxidative stress response [114], whereas inhibition of S-ALP, S-UE, and S-SC suggests that microbial capacity to provision nutrients to plants was compromised [30,115].

#### 4.3.3. Plant Physiological Responses Under Combined Stress

The deterioration of the soil environment and the disruption of nutrient supply were ultimately transmitted to and exacerbated physiological disorders in the plants. In the antioxidant system, increased POD activity (Figure 1), which, together with an insufficient ROS scavenging capacity, resulted in elevated MDA content (Figure 1). This indicates that membrane lipid peroxidation damage could not be mitigated, as microbial-mediated support for plant antioxidant systems was diminished under combined stress [116]. Simultaneously, Pro content was decreased (Figure 1), suggesting a potential impairment of its biosynthetic pathway [117], potentially linked to reduced microbial supply of precursors or signaling molecules. Marginal increases in total chlorophyll content and chlorophyll a/b ratio (Figure 1) align with inhibited chlorophyll synthesis and PSII impairment, rather than healthy physiological adjustment [118,119].

Collectively, the inhibitory effect of saline-alkali combined stress on *Q. mongolica* seedlings arises from synergistic disruption of the plant–soil–microbe continuum, fundamentally distinct from the hormetic responses under single stressors. Combined stress imposes dual pressures of high Na^+^ toxicity and elevated pH that overwhelm regulatory capacity, shifting energy balance from growth investment to damage repair. The interaction of stressors amplifies negative effects beyond the sum of individual stresses, revealing woody plant vulnerability to complex saline-alkali environments. This highlights the need to prioritize the management of either salinity or alkalinity individually in afforestation practices to avoid synergistic inhibition.

## 5. Conclusions

This study reveals that *Q. mongolica* responds to saline-alkali stress through coordinated rhizosphere–microbe–plant interactions. Under single salt or alkali stress, beneficial microorganisms such as *Altererythrobacter* under salt stress and *Sphingomonas* and *Devosia* under alkali stress were enriched, maintaining soil enzyme activities and activating antioxidant and osmotic systems to promote growth. Under combined stress, however, these microbial groups persisted but functional decoupling occurred, with enzyme activities declining and physiological regulation failing, leading to oxidative damage and growth inhibition. This decoupling identifies a critical threshold at which microbial enrichment no longer ensures functional maintenance, marked by the synergistic increase in pH and EC. These findings offer a mechanistic basis for defining survival thresholds of *Q. mongolica* and guiding stress management in temperate forest restoration.

## Figures and Tables

**Table 1:** Salinity-alkalinity stress concentration gradient.

Alkaline Stress Treatment Concentration (mmol·L^−1^)	Salt Stress Treatment Concentration (mmol·L^−1^)			
	0	50	100	200
0	CK	S_1_	S_2_	S_3_
50	A_1_	S_1_A_1_	S_2_A_1_	S_3_A_1_
100	A_2_	S_1_A_2_	S_2_A_2_	S_3_A_2_
150	A_3_	S_1_A_3_	S_2_A_3_	S_3_A_3_

## Data Availability

The original contributions presented in this study are included in the article/Supplementary Materials. Further inquiries can be directed to the corresponding author.

## Supplementary Materials

The following supporting information can be downloaded at: https://www.mdpi.com/article/10.3390/microorganisms14030547/s1, Table S1: A two-way ANOVA of Growth and Physiological Indices of *Quercus mongolica* in Response to Saline-Alkali Stress. Table S2: A two-way ANOVA of Rhizosphere Soil Physicochemical Properties and Enzyme Activity Indices in *Quercus mongolica* in Response to Saline-Alkali Stress. Table S3: A two-way ANOVA of Rhizosphere Soil Microbial Diversity Indices in *Quercus mongolica* in Response to Saline-Alkali Stress. Table S4: PLS-PM Variable Correlation Index Values. Figure S1: Conceptual Model Diagram.
