# Supplementary material for: Underlying Mechanisms for Growth Promotion by Low-Concentration Single Salt and Alkali Stresses and Growth Inhibition by Combined Salt-Alkali Stress in Quercus mongolica
Source: Microorganisms. 2026 Feb 27;14(3):547. doi: 10.3390/microorganisms14030547 (PMC13029177; doi:10.3390/microorganisms14030547)
Supplement: Supplementary file 1 [file microorganisms-14-00547-s001.zip › microorganisms-4161706-supplementary.pdf]

Table S1 A two-way ANOVA of Growth and Physiological Indices of *Quercus mongolica* in Response to Saline-Alkali Stress

Factors	Salt stress		Alkaline salt stress		Salt stress×Alkaline salt stress	
	F	P	F	P	F	P
Plant height increment	247.500	***	66.897	0.000	16.541	0.000
Stem diameter increment	4.504	0.000	7.291	0.000	1.088	0.384
Total plant biomass	519.809	0.000	171.828	0.000	15.225	0.000
SOD	112.296	0.000	100.723	0.000	10.531	0.000
POD	517.572	0.000	155.995	0.000	33.828	0.000
CAT	599.703	0.000	50.632	0.000	19.273	0.000
MDA	506.802	0.000	19.392	0.000	68.516	0.000
Pro	43.749	0.000	224.411	0.000	90.724	0.000
SS	228.478	0.000	161.760	0.000	3.670	0.001
SP	205.949	0.000	28.344	0.000	14.313	0.000
Total chlorophyll content	201.546	0.000	248.065	0.000	18.242	0.000
Chlhorophyll a/b ratio	139.213	0.000	171.471	0.000	51.303	0.000

Table S2 A two-way ANOVA of Rhizosphere Soil Physicochemical Properties and Enzyme Activity Indices in *Quercus mongolica* in Response to Saline-Alkali Stress

Factors	Salt stress		Alkaline salt stress		Salt stress×Alkaline salt stress	
	F	P	F	P	F	P
pH	43.982	0.000	788.626	0.000	21.890	0.000
EC	1519.151	0.000	103.949	0.000	85.199	0.000
SWC	498.983	0.000	384.880	0.000	36.002	0.000
TOC	271.022	0.000	138.577	0.000	37.194	0.000
TN	474.006	0.000	4639.075	0.000	1112.820	0.000
S-UE	2322.104	0.000	341.171	0.000	129.559	0.000
S-CAT	185.311	0.000	874.365	0.000	76.962	0.000
S-ALP	538.520	0.000	74.306	0.000	33.165	0.000
S-SC	443.837	0.000	226.825	0.000	53.729	0.000

Table S3 A two-way ANOVA of Rhizosphere Soil Microbial Diversity Indices in *Quercus mongolica* in Response to Saline-Alkali Stress

Factors	Salt stress		Alkaline salt stress		Salt stress*Alkaline salt stress	
	F	P	F	P	F	P
ACE	9.446	0.013	13.791	0.005	/	/
Observed_Species	10.409	0.010	9.605	0.013	/	/
Chao1	3.880	0.080	10.794	0.009	/	/
Shannon	3.126	0.111	9.446	0.167	/	/
Simpson	11.227	0.009	10.794	0.000	/	/

Table S4 PLS-PM Variable Correlation Index Values

Variable Name	AVE	CR	R2
Stress	1	1	/
Soil physical and chemical propeties	1	1	0.627
Soil enzyme activity	1	1	0.781
Microbial diversity and community compositions	0.562	0.712	0.320
Microbial functional groups	0.412	0.428	0.381
Growth response	1	1	0.471
Physical response	0.831	0.908	0.123
Biomass	0.764	1	/

Note: In this study, biomass serves as a single-indicator latent variable. The calculation of its CR contains technical errors. The actual value should be 1.Soil physical and chemical propeties: pH, EC, SWC, TOC, TN. Soil enzyme activity: S-UE, S-ALP, S-CAT, S-SC. Microbial diversity and community compositions: diversity indices, key taxa abundances. Microbial functional groups: metabolic pathway and resistance gene abundances. Physical response: antioxidant enzymes, osmolytes, chlorophyll parameters. Growth response: Plant height increment, Stem diameter increment. Biomass: total plant biomass.

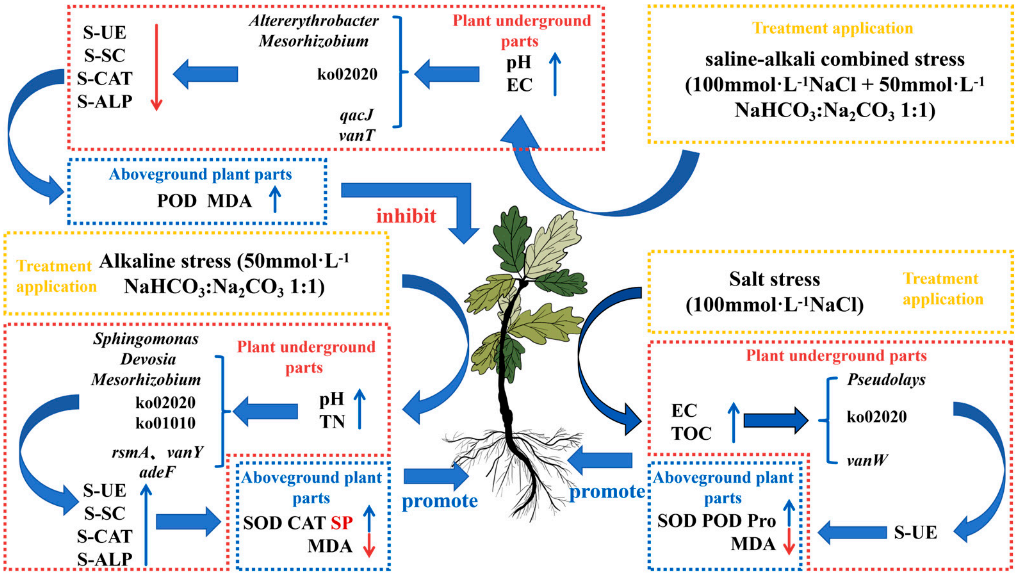

Figure S1 Conceptual Model Diagram
